# Effect of Pharmacogenetic Testing for Statin Myopathy Risk vs Usual Care on Blood Cholesterol: A Randomized Clinical Trial

**DOI:** 10.1001/jamanetworkopen.2020.27092

**Published:** 2020-12-03

**Authors:** Jason L. Vassy, J. Michael Gaziano, Robert C. Green, Ryan E. Ferguson, Sanjay Advani, Stephen J. Miller, Sojeong Chun, Anthony K. Hage, Soo-Ji Seo, Nilla Majahalme, Lauren MacMullen, Andrew J. Zimolzak, Charles A. Brunette

**Affiliations:** 1 VA Boston Healthcare System, Boston, Massachusetts; 2 Department of Medicine, Harvard Medical School, Boston, Massachusetts; 3 Department of Medicine, Brigham and Women’s Hospital, Boston, Massachusetts; 4 Ariadne Labs, Boston, Massachusetts; 5 Department of General Internal Medicine, Boston University School of Medicine, Boston, Massachusetts; 6 Department of Epidemiology, Boston University School of Public Health, Boston, Massachusetts; 7 Massachusetts College of Pharmacy and Health Sciences, Boston; 8 Baylor College of Medicine, Houston, Texas; 9 Michael E. DeBakey VA Medical Center, Houston, Texas

## Abstract

**Importance:**

Nonadherence to statin guidelines is common. The solute carrier organic anion transporter family member 1B1 (*SLCO1B1*) genotype is associated with simvastatin myopathy risk and is proposed for clinical implementation. The unintended harms of using pharmacogenetic information to guide pharmacotherapy remain a concern for some stakeholders.

**Objective:**

To determine the impact of delivering *SLCO1B1* pharmacogenetic results to physicians on the effectiveness of atherosclerotic cardiovascular disease (ASCVD) prevention (measured by low-density lipoprotein cholesterol [LDL-C] levels) and concordance with prescribing guidelines for statin safety and effectiveness.

**Design, Setting, and Participants:**

This randomized clinical trial was performed from December 2015 to July 2019 at 8 primary care practices in the Veterans Affairs Boston Healthcare System. Participants included statin-naive patients with elevated ASCVD risk. Data analysis was performed from October 2019 to September 2020.

**Interventions:**

*SLCO1B1* genotyping and results reporting to primary care physicians at baseline (intervention group) vs after 1 year (control group).

**Main Outcomes and Measures:**

The primary outcome was the 1-year change in LDL-C level. The secondary outcomes were 1-year concordance with American College of Cardiology–American Heart Association and Clinical Pharmacogenetics Implementation Consortium (CPIC) guidelines for statin therapy and statin-associated muscle symptoms (SAMS).

**Results:**

Among 408 patients (mean [SD] age, 64.1 [7.8] years; 25 women [6.1%]), 193 were randomized to the intervention group and 215 were randomized to the control group. Overall, 120 participants (29%) had a *SLCO1B1* genotype indicating increased simvastatin myopathy risk. Physicians offered statin therapy to 65 participants (33.7%) in the intervention group and 69 participants (32.1%) in the control group. Compared with patients whose physicians did not know their *SLCO1B1* results at baseline, patients whose physicians received the results had noninferior reductions in LDL-C at 12 months (mean [SE] change in LDL-C, −1.1 [1.2] mg/dL in the intervention group and −2.2 [1.3] mg/dL in the control group; difference, −1.1 mg/dL; 90% CI, −4.1 to 1.8 mg/dL; *P* < .001 for noninferiority margin of 10 mg/dL). The proportion of patients with American College of Cardiology–American Heart Association guideline-concordant statin prescriptions in the intervention group was noninferior to that in the control group (12 patients [6.2%] vs 14 patients [6.5%]; difference, −0.003; 90% CI, −0.038 to 0.032; *P* < .001 for noninferiority margin of 15%). All patients in both groups were concordant with CPIC guidelines for safe statin prescribing. Physicians documented 2 and 3 cases of SAMS in the intervention and control groups, respectively, none of which was associated with a CPIC guideline–discordant prescription. Among patients with a decreased or poor *SLCO1B1* transporter function genotype, simvastatin was prescribed to 1 patient in the control group but none in the intervention group.

**Conclusions and Relevance:**

Clinical testing and reporting of *SLCO1B1* results for statin myopathy risk did not result in poorer ASCVD prevention in a routine primary care setting and may have been associated with physicians avoiding simvastatin prescriptions for patients at genetic risk for SAMS. Such an absence of harm should reassure stakeholders contemplating the clinical use of available pharmacogenetic results.

**Trial Registration:**

ClinicalTrials.gov Identifier: NCT02871934

## Key Points

**Question:**

Can pharmacogenetic results for statin myopathy risk be used clinically without the unintended harms of statin avoidance or underdosing?

**Findings:**

In this randomized clinical trial including 408 patients, statin-naive patients whose physicians knew their *SLCO1B1* genotype results at baseline did not have poorer low-density lipoprotein cholesterol reductions after 1 year, compared with patients who received usual care.

**Meaning:**

Although these findings do not support the widespread adoption of stand-alone preemptive *SLCO1B1* genotype testing, they may allay stakeholder concerns about the potential unintended harms of the clinical use of such information.

## Teaser

This randomized clinical trial examines the impact of delivering *SLCO1B1* pharmacogenetic results to physicians on low-density lipoprotein cholesterol levels and concordance with prescribing guidelines for statin safety and effectiveness.

## Introduction

Nearly all patients carry 1 or more genetic variants deemed actionable for their association with either the effectiveness or safety of at least 1 medication.^1,2^ High-quality evidence for such pharmacogenetic associations derives from decades of knowledge about candidate genes involved in pharmacokinetic pathways and from more recent developments in genome-wide association studies and large-scale phenotyping of drug response.^3^ However, validation of these drug-gene associations alone is insufficient to demonstrate whether the clinical use of that information is associated with improved patient outcomes. The absence of such outcomes data remains a barrier to the adoption of pharmacogenetic testing by health care practitioners, health systems, and payers. Indeed, the US Food and Drug Administration (FDA) has warned laboratories to stop marketing certain pharmacogenetic tests that it has not reviewed for safety and effectiveness, citing concerns that physicians and patients will change drug therapy on the basis of such results, potentially leading to incorrect treatment and serious health consequences.^4,5^

One well-described pharmacogenetic association is that between the solute carrier organic anion transporter family member 1B1 (*SLCO1B1*) gene and statin-associated muscle symptoms (SAMS). Statins, or 3-hydroxy-3-methylglutaryl-CoA reductase inhibitors, are cholesterol-lowering medications used by millions of patients for the primary and secondary prevention of atherosclerotic cardiovascular disease (ASCVD).^6^ In 2008, a genome-wide association study^7^ identified an association between the common nonsynonymous c.521T>C variant in *SLCO1B1* (rs4149056) and severe simvastatin-related myopathy, and subsequent studies^8,9,10^ have reported an association between this variant and milder phenotypes of statin intolerance. The association between this genetic variant and SAMS appears strongest for simvastatin specifically; as a result, the Clinical Pharmacogenetics Implementation Consortium (CPIC) has published guidelines for simvastatin prescribing and dosing when a patient’s *SLCO1B1* genotype is known.^11^ However, whether integrating *SLCO1B1* testing into routine clinical care improves patient outcomes is unknown.^12^ Of particular relevance to statins is the question of whether pharmacogenetic results might influence physician and patient behavior around initiation of and adherence to therapy, given that concordance with recommended guidelines is suboptimal in many real-world clinical settings.^13,14,15^ In an era when patients increasingly have information about their genetic make-up, including *SLCO1B1* genotype, from clinical or commercial sources, it might be more important to demonstrate that the clinical use of that information does not have the unintended consequence of worsening ASCVD prevention efforts than to demonstrate that it prevents simvastatin myopathy.

We conducted a noninferiority randomized clinical trial to test the primary hypothesis that *SLCO1B1* genotyping among statin-naive primary care patients with ASCVD risk factors does not worsen 12-month reductions in low-density lipoprotein cholesterol (LDL-C). Prespecified secondary outcomes included 12-month concordance with CPIC guidelines for simvastatin use, concordance with statin guidelines for ASCVD prevention, and physician-documented SAMS.

## Methods

### Study Design and Oversight

The Integrating Pharmacogenetics in Clinical Care (I-PICC) Study was a pragmatic randomized clinical trial comparing the delivery of *SLCO1B1* pharmacogenetic results to primary care physicians vs usual care. Detailed descriptions of the trial design, pragmatic elements, and recruitment and enrollment have been published previously.^16,17^ The Veterans Affairs (VA) Boston Healthcare System institutional review board approved this study. The trial protocol is provided in Supplement 1. This study follows the Consolidated Standards of Reporting Trials (CONSORT) reporting guideline.

### Setting

The I-PICC Study enrolled physicians and patients across 8 primary care practices in the VA Boston Healthcare System in eastern Massachusetts. Patient enrollment occurred from December 2015 to July 2018, and all patients were followed up for 1 year, through July 2019.

### Participants

All primary care physicians at the 8 locations were eligible to participate. Patient eligibility criteria were assignment to an enrolled physician, age 40 to 75 years, absence of prior statin prescription confirmed both by medical record review and patient telephone call, and at least 1 of the following ASCVD risk factors specified in the American College of Cardiology–American Heart Association (ACC-AHA) guidelines: prior ASCVD, diabetes, LDL-C level greater than or equal to 190 mg/dL (to convert to millimoles per liter, multiply by 0.0259), or 10-year ASCVD risk greater than or equal to 7.5%.^18^

### Recruitment, Enrollment, and Randomization

After brief presentations at staff meetings, physicians gave written informed consent for their own participation through the electronic health record (EHR).^16^ Patients gave oral consent to participation by telephone call with the study staff but were not enrolled unless and until they underwent a blood draw as part of their routine clinical care. A daily semiautomated electronic query alerted study staff each time the clinical laboratory received a whole-blood specimen for a consented patient (eg, for complete blood count or hemoglobin A_1c_ testing), at which time the study staff forwarded a laboratory order for *SLCO1B1* genotyping through the EHR as a clinical alert to the enrolled physician for signature. The physician’s signature of the laboratory order enrolled the patient in the study. Upon signature of the laboratory order, the extant blood sample was sent to a reference laboratory (Boston Heart Diagnostics, Framingham, MA) for *SLCO1B1* rs4149056 genotyping, and the participants were randomly allocated to having their *SLCO1B1* results delivered to their physician at baseline (intervention group) or after 12 months (control group).

### Intervention

The *SLCO1B1* results were entered as structured data in the EHR after a median (interquartile range) turnaround time of 8 (7-9) days after enrollment of each patient in the intervention group. The results screen included genotype and standardized terms for drug transporter function phenotype (T/T, normal function; T/C, decreased function; or C/C, poor function)^19^ and CPIC recommendations for the use and dosing of simvastatin when *SLCO1B1* genotype is known.^11^ A clinical alert notified the ordering physician when the results were reported in the EHR. The patient’s calculated 10-year ASCVD risk or other potential indication for statin therapy was not explicitly communicated to the physician. Because this trial endeavored to model routine medical practice, study staff members themselves did not send the *SLCO1B1* results directly to patients during the observation period, but an optional *SLCO1B1* results and interpretation letter template was available in the EHR for physicians to communicate results to their patients.^16^ For patients allocated to the control group, physicians received no further communication from the study staff after patient enrollment until the end of the 12-month observation period, at which time their *SLCO1B1* results were delivered to their physicians through the EHR.

### Outcomes

#### Primary Outcome

Data on outcomes were collected from the VA corporate data warehouse,^20^ EHR review, and a brief end-of-study patient telephone survey, as described elsewhere.^16^ The primary outcome was change in LDL-C, defined as the most recent LDL-C value on or before the enrollment date subtracted from the most recent LDL-C value 12 months after enrollment. Baseline LDL-C values were carried forward for any patient who did not undergo repeated LDL-C testing during the observation period.

#### Secondary Outcomes

The study had 3 prespecified secondary outcomes, as described elsewhere^16^: (1) concordance with CPIC guidelines for simvastatin use, determined by comparing each participant’s *SLCO1B1* genotype and statin type and dose 12 months after enrollment^11^; (2) concordance with ACC-AHA guidelines for ASCVD prevention, determined by comparing each participant’s ASCVD risk profile with the intensity of their statin therapy 12 months after enrollment^18^; and (3) physician-documented SAMS during the 12-month observation period, determined from medical record review of all patient notes during the 12 months after enrollment. Additional prespecified exploratory outcomes included initiation of and changes to statin therapy during the 12-month observation period; patient continuous medication adherence to statin therapy, derived from pharmacy data and defined as proportion of days covered by medication possession greater than or equal to 80%^21,22^; recall of genetic testing and results; and patient-perceived necessity of and concerns about medications.^23^

### Statistical Analysis

Statistical analysis was conducted using SAS statistical software version 9.4 (SAS Institute). Outcomes were analyzed with an intention-to-treat approach by randomization group. For the primary outcome of 12-month change in LDL-C, we used generalized estimating equations (GEEs)^24^ accounting for clustering by physician to derive marginal mean estimates. By use of a noninferiority design, GEEs tested the primary null hypothesis that *SLCO1B1* testing resulted in poorer 12-month LDL-C reductions compared with no testing by a prespecified margin of greater than 10 mg/dL, chosen for its association with a reduction in 5-year ASCVD risk of 5%.^25^ We used GEEs assuming independence to test the null hypothesis that the proportion of patients in the control group whose prescriptions at 12 months met ACC-AHA guidelines for ASCVD prevention was better by a noninferiority margin of 15% compared with the intervention group. Ninety percent confidence intervals for noninferiority testing, corresponding to a 1-sided α = .05, were based on GEE estimates of the difference in outcomes between groups and their robust SEs. We estimated Fisher exact tests to test the null hypotheses that the proportion of patients with CPIC guideline concordance and with SAMS 12 months after enrollment did not differ between the 2 groups, using a superiority design. A sample size of 408 total patients enabled 80% or higher power at a 1-sided α = .05 to exclude a between-group noninferiority margin of 10 mg/dL in the primary outcome of LDL-C 12 months after enrollment and 80% or higher power at a 2-sided α = .05 to detect a between-group difference of 15% in the secondary outcome of CPIC guideline concordance. Exploratory outcomes are presented with descriptive statistics without hypothesis testing. Data analysis was performed from October 2019 to September 2020.

## Results

### Participant Characteristics

**Figure 1.**
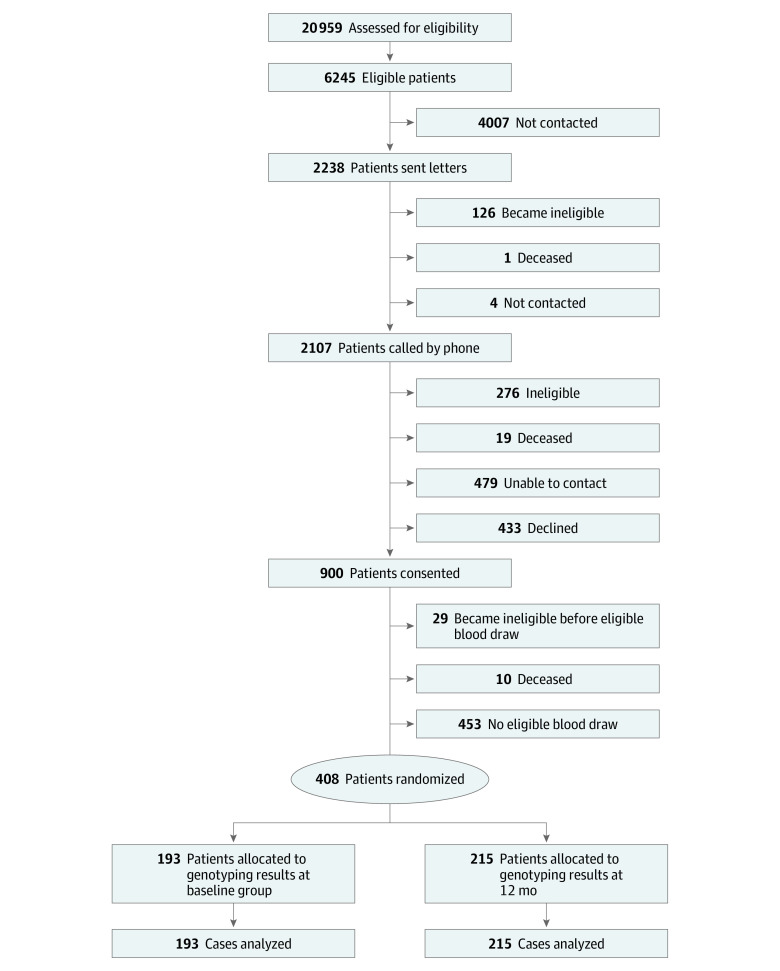
Patient Flowchart for the Integrating Pharmacogenetics in Clinical Care Study

Enrollment and randomization of the prespecified sample size of 408 patients, cared for by 47 physicians, was completed on July 17, 2018 (Figure 1).^17^ The mean (SD) age of the participants was 64.1 (7.8) years, 25 (6.1%) were women, 56 (13.7%) were non-White, and 8 (2.0%) were of Hispanic or Latino ethnicity (Table 1). Of the patients, 193 were randomized to the intervention group (with genotyping results known at baseline), and 215 were randomized to the control group with (genotyping results unknown at baseline). Overall, 98 patients (24.0%) had diabetes and 98 (24.0%) had prior ASCVD; 223 patients (54.7%) were potentially eligible for statin therapy only because they had 10-year ASCVD risk of 7.5% or higher. Overall, 120 participants (29%) had a *SLCO1B1* genotype indicating increased simvastatin myopathy risk (T/C or C/C genotype) (Table 1).

**Table 1.** Baseline Characteristics of Integrating Pharmacogenetics in Clinical Care Study Participants

Characteristic	Participants, No. (%)	
	Genotyping results known at baseline (n = 193)	Genotyping results known at 12 mo (n = 215)
Age, mean (SD), y	64.2 (7.8)	63.9 (7.7)
Women	9 (4.7)	16 (7.4)
Non-White race^a^	30 (15.5)	26 (12.1)
Hispanic or Latino ethnicity^a^	2 (1.0)	6 (2.8)
Smokers	59 (30.6)	78 (36.3)
Meeting ACC-AHA statin criteria^b^		
ASCVD	52 (26.9)	46 (21.4)
LDL-C >190 mg/dL	5 (2.6)	6 (2.8)
Diabetes	47 (24.4)	51 (23.7)
10-y ASCVD risk ≥7.5%	171 (88.6)	196 (91.2)
*SLCO1B1* genotype		
Normal function (T/T)	148 (76.7)	140 (65.1)
Decreased function (T/C)	40 (20.7)	70 (32.6)
Poor function (C/C)	5 (2.6)	5 (2.3)

Abbreviations: ACC-AHA, American College of Cardiology–American Heart Association; ASCVD, atherosclerotic cardiovascular disease; LDL-C, low-density lipoprotein cholesterol.

SI conversion factor: To convert LDL-C to mmol/L, multiply by 0.0259.

^a^ Race and ethnicity were collected from administrative data to assess generalizability of enrolled cohort to overall health care system population.

^b^ Categories sum to greater than 100% because criteria are not mutually exclusive (see text).

### Statin Prescriptions

During the study, physicians documented offering statin therapy to 65 participants (33.7%) in the intervention group and 69 participants (32.1%) in the control group, among whom 42 (21.8% of total) and 50 (23.3% of total) declined, respectively (Table 2). Statin therapy was prescribed at some time during the 12-month study period for 26 patients (13.5% of total) in the intervention group and 24 patients (11.1% of total) in the control group.

**Table 2.** Statin Prescription Outcomes of the Integrating Pharmacogenetics in Clinical Care Study

Outcome	Participants, No. (%)	
	Genotyping results known at baseline (n = 193)	Genotyping results known at 12 mo (n = 215)
Statin offered by physician	65 (33.7)	69 (32.1)
Statin declined^a^	42 (64.6)	50 (72.5)
Statin prescribed^a^	26 (40.0)	24 (34.8)
Statin adherence^b^	9 (45.0)	9 (45.0)
Statin discontinued	3 (11.5)	4 (16.7)
ACC-AHA concordance at 12 mo^c^	12 (6.2)	14 (6.5)
CPIC concordance at 12 mo^d^	193 (100.0)	215 (100.0)

Abbreviations: ACC-AHA, American College of Cardiology–American Heart Association; CPIC, Clinical Pharmacogenetics Implementation Consortium.

^a^ Percentages may sum to more than 100% because a patient could both initially decline statin therapy and then be prescribed statin therapy later during the observation period.

^b^ Denotes the number of participants with proportion of days covered by medication possession greater than or equal to 80% from statin initiation through the end of study enrollment; calculable denominators for each group are 20 participants.

^c^ *P* < .001, corresponding to 1-sided noninferiority test assuming margin of 15% favoring control.

^d^ *P* > .99, corresponding to 2-sided test for superiority.

### Primary Outcome

**Figure 2.**
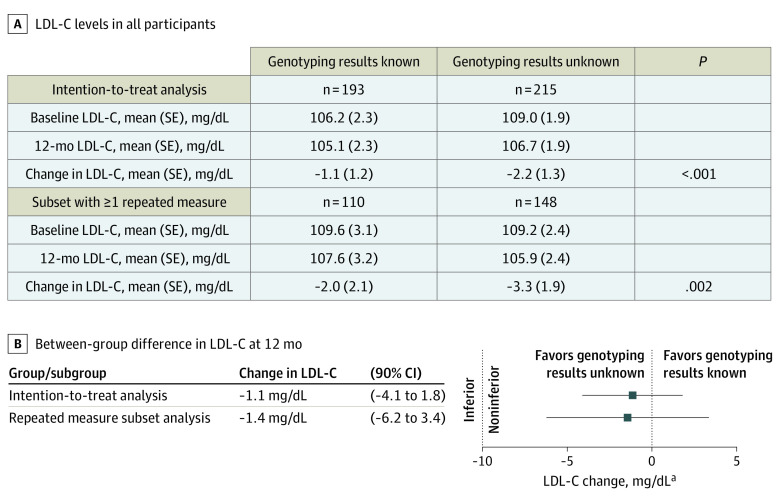
Change in Low-Density Lipoprotein Cholesterol (LDL-C) Values Among Integrating Pharmacogenetics in Clinical Care Study Participants SI conversion factor: To convert LDL-C to mmol/L, multiply by 0.0259.

The mean (SE) LDL-C level at baseline was 106.2 (2.3) mg/dL in the intervention group and 109.0 (1.9) mg/dL in the control group (Figure 2). After 12 months of follow-up, the mean (SE) change in LDL-C was −1.1 (1.2) mg/dL in the intervention group and −2.2 (1.3) mg/dL in the control group. The between-group difference was consistent with the prespecified alternative hypothesis that *SLCO1B1* testing does not worsen LDL-C levels by more than the noninferiority margin of 10 mg/dL, compared with usual care (difference, −1.1 mg/dL; 90% CI, −4.1 to 1.8 mg/dL; *P* < .001). Analysis among the subset of 258 patients with at least 1 repeated LDL-C measurement during the 12 months of follow-up yielded consistent results (difference, −1.4 mg/dL; 95% CI, −6.2 to 3.4 mg/dL; *P* = .002 (Figure 2). Eighty-one patients (42.0%) in the intervention group and 88 patients (40.9%) in the control group had end-of-study LDL-C values less than 100 mg/dL.

### Secondary Outcomes

Twelve months after enrollment, 12 patients (6.2%) in the intervention group and 14 patients (6.5%) in the control group had statin prescriptions that were concordant with ACC-AHA guidelines for statin therapy for ASCVD prevention (difference, −0.003; 90% CI, −0.038 to 0.032; *P* < .001 for noninferiority margin of 15%) (Table 2). All patients in both groups were concordant with CPIC guidelines for genotype-based safe statin dosing at 12 months (difference, 0.0; Fisher exact test *P* > .99) (Table 2). Physicians documented 2 (1.0%) and 3 (1.4%) possible cases of SAMS in the intervention and control groups, respectively (difference, 0.004; Fisher exact test *P* > .99) (eTable 1 in Supplement 2); only 1 of these was associated with simvastatin, prescribed at a dose of 20 mg for a patient with the normal T/T genotype in the intervention group. The physician of another patient in the control group with the decreased *SLCO1B1* transporter function T/C genotype documented possible SAMS with an atorvastatin dose of 20 mg, before the patient or physician knew the genotype results.

**Figure 3.**
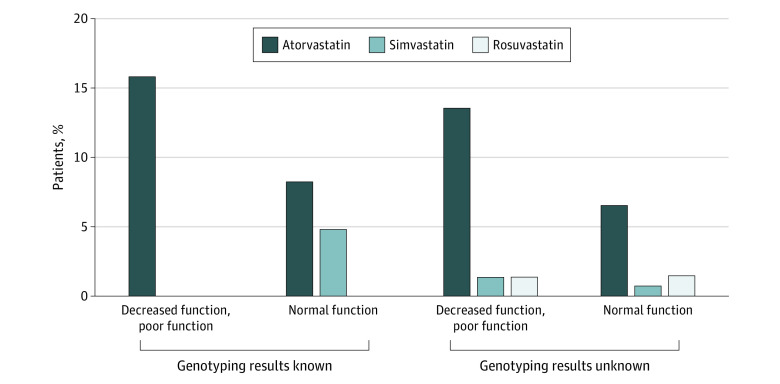
Statin Initiations at 12 Months by Study Group and Genotype Among Integrating Pharmacogenetics in Clinical Care Study Participants With Known and Unknown Genotyping Results Bars represent percentages of patients with a given genotype and study group assignment who were prescribed statin therapy by 12 months. Among patients whose genotyping results were known at baseline, 45 had the genotype for decreased or poor function and 148 had the genotype for normal function. Among patients whose genotyping results were not known at baseline, 75 had the genotype for decreased or poor function and 140 had the genotype for normal function.

Of the 26 statin prescriptions in the intervention group, 7 were for simvastatin, and all of these were for patients with the normal transporter T/T genotype (Figure 3). Atorvastatin was the only statin prescribed to 7 patients in the intervention group with a decreased or poor transporter genotype (T/C or C/C). In contrast, approximately equal numbers of patients with the T/T genotype and with the T/C or C/C genotypes received prescriptions for atorvastatin, rosuvastatin, and simvastatin in the control group, including 1 patient with the T/C genotype who was prescribed simvastatin 20 mg (Figure 3). Among the 50 patients for whom statin therapy was initiated, statin adherence, defined as greater than or equal to 80% of days covered, was achieved by equal numbers in the 2 groups (Table 2).

### Exploratory Outcomes

Among the 193 patients in the intervention group, physicians entered additional documentation about the *SLCO1B1* results in the EHR for 32 patients (16.6%) and documented communicating results to 30 patients (15.5%) during the 12 months after enrollment (eTable 2 in Supplement 2). In the 12-month survey, only 11 patients (6.5%) in the intervention group recalled having undergone a pharmacogenetic test for SAMS risk in the prior year, of whom only 2 correctly recalled the interpretation of their results. At 12 months, patients in the intervention and control groups did not differ in their perceived necessity of and concerns about medications (eTable 3 in Supplement 2).

## Discussion

In this randomized clinical trial, preemptive *SLCO1B1* genotype testing among statin-naive patients was noninferior to no testing in reducing LDL-C and achieving concordance with ASCVD prevention guidelines. No physician prescribed simvastatin to a patient known to have decreased or poor *SLCO1B1* transporter function genotype. Although these results do not support a patient benefit from stand-alone preemptive *SLCO1B1* genotyping, they help allay concerns about the potential unintended harms of using such pharmacogenetic results in medical practice if they are available.

Many health care systems have launched pharmacogenetic testing programs, often in the context of research studies or clinical innovation demonstration projects.^26,27,28,29,30,31,32,33,34^ Most of these endeavors have chosen to implement some number of well-validated drug-gene associations, such as clopidogrel-*CYP2C19* and codeine-*CYP2D6*.^35,36^ Still, regulatory uncertainty remains a barrier to more widespread uptake. The FDA has expressed concern that some pharmacogenetic tests lack validity and that using the results to alter drug treatment could “lead to immediate serious health consequences for patients.”^5^ Although clinical laboratory, molecular pathology, and pharmacogenetics professional societies have disagreed with the agency’s assertions,^37,38^ empirical demonstration that the clinical use of pharmacogenetic test results does not worsen patient outcomes will help inform this debate.

In the context of statin treatment, given their demonstrated effectiveness in primary and secondary ASCVD prevention and the association of poor statin adherence with adverse ASCVD outcomes,^25,39,40,41,42^ pharmacogenetic testing would cause unintentional harm if it paradoxically made patients less likely to initiate and adhere to therapy with any statin, including simvastatin.^16^ Even without pharmacogenetic testing, physician and patient behavior around statin therapy is already highly variable, and many patients remain hesitant to adhere to recommendations.^13,14,15^ Change in LDL-C represents a common clinical end point for these variable physician and patient behaviors. We found that *SLCO1B1* testing was not associated with a between-group difference in LDL-C reduction outside the noninferiority limit of 10 mg/dL, a surrogate outcome for a reduction in 5-year ASCVD risk of 5%.^25^ A previous randomized trial among 159 previously statin-intolerant patients found that *SLCO1B1* genotyping and reporting, compared with end-of-study reporting, resulted in more new statin reinitiations and lower LDL-C levels 3 months after enrollment.^43^ Together, these findings provide some reassurance about possible unintended harms of using *SLCO1B1* results.

### Limitations

Pragmatic trials combine the rigor of randomization and enhanced generalizability to real-world medical practice,^44^ but they introduce limitations evidenced in this study. First, fewer enrollees than expected were prescribed statin therapy generally and simvastatin therapy specifically during the observation period, likely the result of patient reluctance and physician prescribing patterns that target statin therapy to a goal LDL-C less than 100 mg/dL instead of to ASCVD risk categories, particularly among patients meeting statin eligibility only because of the more recently recommended criterion of 10-year ASCVD risk greater than or equal to 7.5%. A treatment trial with a protocolized genotype-guided prescribing algorithm would have ensured higher rates of statin initiation and increased the power to demonstrate superiority of *SLCO1B1* testing. Second, the absence of protocolized LDL-C measurements at baseline and follow-up introduces the potential for bias in the primary outcome, although analyses among those with at least 1 repeated LDL-C measurement yielded results similar to those for the intention-to-treat analyses. Third, by chance, randomization resulted in a lower proportion of patients with decreased or poor *SLCO1B1* transporter function genotypes in the intervention group than in the control group. Stratified randomization would have prevented this imbalance, the impact of which on the trial results is unknown but expected to be minimal, because physicians and patients in the control group likely proceeded with usual care, blinded to genotype. Fourth, the pragmatic design may have limited physician and patient engagement with the pharmacogenetic results. A less pragmatic trial with dedicated study visits and a less subtle delivery of pharmacogenetic test results to physicians and patients might have resulted in greater engagement with the intervention and potentially greater clinical impact. Our observation that only 15.5% of physicians documented communicating *SLCO1B1* results to intervention patients is an imperfect measurement of that engagement. Guidelines also recommend shared decision-making between patients and physicians about statin therapy, which was not measured in this trial.

## Conclusions

In this practical randomized clinical trial, the clinical integration of *SLCO1B1* pharmacogenetic testing for simvastatin myopathy risk did not result in poorer measures of ASCVD prevention in routine primary care settings. Such an absence of harm may reassure stakeholders contemplating the clinical use of pharmacogenetic information.

## Notes

Supplement 1. **Trial Protocol**

Supplement 2. **eTable 1.** Evidence of Statin-Associated Muscle Symptoms (SAMS) in the Integrating Pharmacogenetics in Clinical Care (I-PICC) Study **eTable 2.** Electronic Health Record (EHR) Documentation and Patient Communication Process Outcomes Among I-PICC Study Patient Participants in the Intervention (PGx+) Arm **eTable 3.** Patient Beliefs About Medications and Test Results Recall at 12 Months

Supplement 3. **Data Sharing Statement**
