# Supplementary material for: Effect of Pharmacogenetic Testing for Statin Myopathy Risk vs Usual Care on Blood Cholesterol: A Randomized Clinical Trial
Source: JAMA Netw Open. 2020 Dec 3;3(12):e2027092. doi: 10.1001/jamanetworkopen.2020.27092 (PMC7716196; doi:10.1001/jamanetworkopen.2020.27092)
Supplement: Supplement 1. — Trial Protocol [file jamanetwopen-e2027092-s001.pdf]

Title: **Clinical safety and efficacy of pharmacogenetics in Veteran care**
Principal Investigator: **Jason L. Vassy**

(1) **Rationale**

(a) Statement of the Problem.

There is great hope that precision medicine will improve patient outcomes in a variety of clinical settings. In particular, pharmacogenetics may be one of the first areas where genomic information finds widespread clinical utility. The goal of pharmacogenetics is to improve the risk-benefit ratio of pharmacotherapy. That is, many believe that using genotype to tailor drug choice and dose will improve efficacy and minimize adverse effects. In fact, large health networks such as the Mayo Clinic and Geisinger Health System are already implementing pharmacogenetic testing and prescription decision support in their electronic health records (EHRs) for patient care. The VA currently uses pharmacogenetic testing in a limited number of specialized clinical settings. It unknown how the introduction of pharmacogenetic testing for a medication used commonly across VA might impact clinical outcomes within the health system.

(b) Hypotheses or Key Question.

This randomized-controlled trial (RCT), also referred to as the Integrating Pharmacogenetics In Clinical Care (I-PICC) Study, will determine the impact of the clinical integration of testing for a well-characterized pharmacogenetic association: the *SLCO1B1* rs4149056 variant associated with simvastatin-induced muscle damage (myopathy). Specifically, it will determine the impact of *SLCO1B1* testing on safe and effective statin prescribing, the occurrence of simvastatin-related myopathy, and patient low-density lipoprotein (LDL) cholesterol levels.

(c) Specific Objectives.

This RCT has two primary aims:

Aim 1 (Drug safety): To determine the impact of *SLCO1B1* pharmacogenetic testing on concordance with pharmacogenetic guidelines for safe simvastatin prescribing and on the incidence of statin-related myopathy in VA (drug safety).

Hypothesis 1: After one year, compared to Veterans not receiving *SLCO1B1* pharmacogenetic testing (PGx), Veterans receiving pharmacogenetic testing (PGx+) will be more likely to meet pharmacogenetics-guided simvastatin dosing guidelines and will have lower incidence of statin-related myopathy.

Aim 2 (Cardiovascular disease, CVD, prevention): To determine the impact of *SLCO1B1* pharmacogenetic testing on LDL cholesterol levels and concordance with CVD prevention guidelines.

Hypothesis 2: After one year, PGx+ and PGx- Veterans will not differ in mean LDL cholesterol levels or in the proportion meeting American College of Cardiology/American Heart Association (ACC/AHA) guidelines for CVD prevention.

(2) **Background and Significance**

(a) Background.

i. Statins and CVD prevention: CVD is the leading cause of death, impaired quality of life, and increased medical costs in the United States[1,2], and Veterans may have even greater CVD risk burden than the general population[3,4]. Epidemiologic studies have shown a direct relationship between LDL cholesterol levels and CVD risk[5,6], and the LDL-lowering HMG-CoA reductase inhibitor medications (statins) have an established role in the primary and secondary prevention of CVD events and mortality[7,8]. As a result, they are widely recommended for many patient populations. In 2013, the ACC/AHA endorsed guidelines that recommended prescribing statins of specific intensities (moderate or high) for 4 patient population: 1) Clinical CVD (acute coronary syndromes, myocardial infarction, stable angina, coronary or other arterial revascularization, stroke, transient ischemic attack, or peripheral arterial disease); 2) Age ≥21 and LDL ≥190 mg/dL; 3) Age 40-75 with diabetes and LDL 70-189 mg/DL; and 4) Age 40-75 without diabetes but 10-year CVD risk ≥7.5%[9]. For this last category, the ACC/AHA recommends that CVD risk be estimated by pooled cohort equations based on five population-based cohorts of African-American and white men and women.

ii. Statins and adverse events: Despite the importance of statins for CVD prevention, poor adherence to statin therapy remains high among patients, including Veterans[10-14], and is associated with increased mortality[15,16]. Reasons for statin non-adherence include their side effects[12,16]. Rhabdomyolysis is an exceedingly rare but potentially life-threatening muscle injury[17] that occurs in 1 in 10,000 patients taking statins[18-20]. This risk is greater with certain statins and increases with dose[21]; accordingly, the FDA recommends against the initiation of treatment with 80 mg of simvastatin daily[20]. More commonly, patients on statins experience milder non-specific muscle pains. These subjective symptoms are experienced by 5-20% of patients taking statins[11,22], although RCT data suggest they do not occur with statins more than with placebo[23]. Indeed, many patients who have previously discontinued statins due to side effects can likely be safely restarted on statin therapy without recurrence of muscle pains[11,24]. Although no universally accepted classification scheme exists, statin-related myopathy can be organized into 3 classes: 1) mild: muscle pains (myalgias) without creatine kinase (CK) elevation; 2) moderate: myalgias with mild CK elevation (<10x the upper limit of normal, ULN); and 3) severe: myalgias with CK≥10x ULN[21,25]. Severe myopathy includes rhabdomyolysis.

iii. Statin myopathy pharmacogenetics: A patient's risk of statin myopathy is increased by certain factors including type and dose of statin, advanced age, and interactions with other drugs such as amiodarone, cimetidine, clarithromycin, and azole anti-fungal medications[26]. Patient genotype also mediates some of this risk. A genome-wide association study (GWAS) in the SEARCH trial identified a robust association between a common genetic variant (rs4149056) in the *SLCO1B1* gene and simvastatin-related myopathy, an outcome encompassing "definite myopathy" (muscle symptoms with CK levels >10x ULN) and "incipient myopathy" (CK level both >3x ULN and >5x the baseline level and an alanine aminotransferase (ALT) level >1.7x the baseline value without an elevated ALT level alone at any other visit, irrespective of presence or absence of muscle symptoms)[27]. The *SLCO1B1* gene, a member of the OATP/SLCO superfamily of transmembrane transporters, encodes the liver-specific OAT1B1 transporter that regulates statin uptake from the blood into hepatocytes[28,29]. In the SEARCH trial, myopathy occurred among 98 in 6031 patients taking simvastatin 80 mg over a mean of 6 years. The rs4149056 variant in *SLCO1B1* is a well characterized functional non-synonymous valine→alanine change at position 174, associated with statin metabolism. SEARCH found that each copy of the C allele in rs4149056 in *SLCO1B1* increased the risk of myopathy by a factor of 4.5 (95% CI 2.6, 7.7); CC homozygotes had a 16.9-fold (95% CI, 4.7 to 61.1) increased risk [27]. This association was replicated in the independent Heart Protection Study, which had randomized patients to placebo vs. simvastatin 40 mg[27]. Further replication among patients in the Go-DARTS Study also found that the functional rs4149056 variant was associated with the milder phenotype of statin intolerance, a composite endpoint of muscle and liver laboratory abnormalities and a change in statin therapy (a different statin, a lower dose, or discontinuation)[30]. The association between the C allele in rs4149056 and myopathy appears strongest for simvastatin, may or may not

be present with atorvastatin, and probably does not occur with pravastatin[20,31-33]. In the open-label STRENGTH study, which randomized patients to receive atorvastatin, simvastatin, or pravastatin, the rs4149056 variant was associated with the composite endpoint of premature statin discontinuation, myalgias, or CK level >3x ULN; in stratified analyses, this association was only significant among patients taking simvastatin[34]. The rs4149056 C allele is common. A 2010 review reported a minor allele frequency (MAF) of 15-20% in individuals with European ancestry, 1-4% in African-Americans, and 6-19% in Asians[35]. The MAF of rs4149056 C is 9% in the 1000 Genomes cohort and ranges from 3% (Africans) to 21% (Finnish) in the populations indexed in the Exome Aggregation Consortium (ExAC) browser, consisting of genotypes from 60,706 individuals[36]. Other genetic loci have been postulated to be associated with statin myopathy, including variants in the *CYP2D6*, *CYP3A4*, *CYP3A4*, *ABCB*, and *ABCG2* genes and the mitochondrial gene *GATM*[37]. However, a recent review found inconsistent evidence between variants in these genes and statin-related myopathy, finding that only the rs4149056 variant in *SLCO1B1* had a strong and consistent association with the condition, particularly for simvastatin[37].

iv. Clinical implementation of *SLCO1B1* genotyping: Some large health systems are incorporating *SLCO1B1* pharmacogenetics into patient care and clinical decision support in their EHRs, including members of the Electronic Medical Records and Genomics (eMERGE) Network[20,38-40] like Vanderbilt University Medical Center, Geisinger Health System, and the Mayo Clinic. Since 2009, the Clinical Pharmacogenetics Implementation Consortium (CPIC), a joint initiative between the Pharmacogenomics Knowledgebase (PharmGKB) and the Pharmacogenomics Research Network (PGRN), has published guidelines for the use and management of specific PGx tests in clinical care. In 2014, CPIC published updated recommendations for simvastatin prescribing when a patient's genotype at *SLCO1B1* rs4149056 is known[20,33]. In particular, these guidelines recommend against simvastatin 40 mg or 80 mg for patients carrying at least one copy of the C allele. Given the inconsistent evidence for the association between *SLCO1B1* and myopathy from statins other than simvastatin and for the association between statin myopathy and genetic loci other than *SLCO1B1* rs4149056, CPIC limits its recommendations only to simvastatin and the rs4149056 genotype. Several Clinical Laboratory Improvement Amendments (CLIA)-certified laboratories across the country, including Boston Heart Diagnostics in Framingham, MA, offer clinical-grade polymerase chain-reaction (PCR) assays for the *SLCO1B1* rs4149056 genotype. However, there is currently equipoise as to whether the use of such testing improves patient outcomes in a health system.

(b) Significance.

Despite the growing implementation of *SLCO1B1* rs4149056 genotyping in health systems across the United States, none of these centers is using a RCT design to evaluate the impact of *SLCO1B1* testing on clinical outcomes. This study will use a randomized design to determine the impact on important patient outcomes, including statin prescribing, LDL cholesterol, and statin-related myopathy. In addition, by enrolling statin-naïve patients at the clinical moment when their providers order a cholesterol panel, this trial will capture a moment of clinical decision-making when *SLCO1B1* rs4149056 genotype might be most clinically relevant.

(c) Relevance to Veterans Health.

CVD is the leading cause of mortality among Veterans, and statins have an established role in CVD risk reduction. Still, many Veterans cannot tolerate statins because of muscle-related adverse effects, which can be partially explained by genetic factors. Pharmacogenetic testing may help guide safer but equally effective statin use in the care of Veterans, but this possibility remains theoretical, absent empiric RCT data. This research will contribute evidence for VHA as it weighs whether to incorporate pharmacogenetic testing for statin myopathy, and genome sequencing more broadly, into patient care.

(3) **Work Proposed**

Total subjects expected to enroll: 408

(a) Timeline.

July 2016 – Dec 2018: Patient recruitment and enrollment at 163 patients/year
Jan 2018 – Mar 2020: 12-month outcome data collection from EHR and patient surveys
Apr 2020 – Dec 2020: Data analysis and presentation/publication of results

Total patients expected to enroll: 408
Total providers expected to enroll: 70

(b) Trial overview and aims (I-PICC Study): This study is an RCT of immediate *SLCO1B1* genotype reporting vs. delayed reporting in Primary Care and Women's Health in the VA Boston Healthcare System, designed to determine the impact of *SLCO1B1* testing on patient outcomes after one year. In keeping with other trials across VA that capitalize on its learning health systems, this trial presents minimal burden to providers and patients through its integration into routine clinical care. Providers will give informed consent for their own participation by signing an informed consent form within a fake patient's electronic medical record in the Computerized Patient Record System (CPRS) or by signing a paper copy of the informed consent form. Once a provider enrolls, the study staff will mail an informed consent letter to his/her eligible statin-naïve patients, describing the study and giving the patients the option to call the study staff to consent to study participation. If a patient does not call the study staff within 10 days of the letter being sent out, the study staff may call the patient to ask if he/she has any questions about the study and whether he/she consents to participating. A patient is not enrolled, however, unless and until the patient's provider signs a laboratory order for *SLCO1B1* testing for that patient. Enrolled patients will be randomized to have their provider receive their *SLCO1B1* results immediately (PGx+) vs. at the end of the 12-month observation period (PGx-). The *SLCO1B1* results will be delivered to the ordering provider as a view alert in the Computerized Patient Record System (CPRS). This 12-month period without *SLCO1B1* results models current standard of care (that is, the absence of genotype information in statin prescribing). One year after enrollment, the study staff will query VA clinical and pharmacy data for the outcomes of interest: myopathy and concordance with CPIC simvastatin guidelines (drug safety) and LDL levels and concordance with ACC/AHA statin guidelines (CVD prevention). The study staff will also call each enrolled patient for a brief telephone survey. Each of these steps in the study protocol is described in greater detail in the Procedures section below.

The trial has two primary aims:

1. Drug safety: To determine the impact of *SLCO1B1* PGx testing on concordance with pharmacogenetic guidelines for safe simvastatin prescribing and on the incidence of statin-related myopathy in VA.

   Hypothesis: After one year, compared to Veterans not receiving *SLCO1B1* PGx testing (PGx-), Veterans receiving pharmacogenetic testing (PGx+) will be more likely to meet CPIC simvastatin guidelines and will have lower incidence of statin-related myopathy.

2. CVD prevention: To determine the impact of *SLCO1B1* pharmacogenetic testing on LDL cholesterol levels and concordance with CVD prevention guidelines.

   Hypothesis: After one year, PGx+ and PGx- Veterans will not differ in mean LDL cholesterol levels or in the proportion meeting 2013 ACC/AHA guidelines for CVD prevention.

(c) Procedures:

i. Provider eligibility, consent, and enrollment: All providers in Primary Care and Women's Health at VA Boston will be eligible to participate. Providers will be educated about the study through presentations at staff meetings and through e-mails and individual outreach. After these educational materials have been presented and distributed, an email will be sent to providers instructing them how to give informed consent if they would like to participate in the study (see attached Initial Provider Recruitment email). Providers have two options for how to give informed consent: through CPRS or on a paper-based form. A 'testpatient' view alert from a fake patient's CPRS record will be sent to each eligible provider by a member of the study team. This 'testpatient' view alert will accompany a progress note containing the text of the provider informed consent (see attached). By signing this 'testpatient' note, the provider is agreeing to enroll in the study as a research subject and allow the study team to contact his/her eligible patients. Study staff will print a paper copy of the provider CPRS consent note to store in the research records of the enrolled providers. For providers who would prefer to sign an informed consent form outside of CPRS, the recruitment email from the study staff will also include the informed consent form as a pdf document, which the provider may print, sign, and return to the study staff by intercampus mail. If a provider does not sign the CPRS order or return a paper copy of the informed consent form within a week, the study staff may send a follow-up email (see attached Follow-up Provider Recruitment email), reminding them about the opportunity to enroll in the study by signing the CPRS alert or returning a signed paper copy of the informed consent form. Provider consent will be tracked by study staff in a secured data file that includes all eligible providers at VA Boston. If and when a provider consents to participate, the study staff will log that he/she is "CONSENTED" and the date and method of consent (CPRS or paper copy). Providers who expressly notify the staff that they are not interested in participating will be designated as "OPTOUT" in this file. All other providers will remain designated as "ELIGIBLE" in the file. Only CONSENTED providers will be considered enrolled in the study, and only the patients of consented providers will be eligible for patient enrollment. After enrolling in this study, a provider may unenroll at any time by contacting the Principal Investigator or study coordinator by phone and request that they no longer receive orders to enroll eligible patients. These providers will be designated as "UNENROLLED" in the provider file.

For this RCT, study staff will enroll up to 70 total providers from Primary Care and Women's Health at VA Boston.

ii. Patient eligibility: The patient eligibility criteria enrich the study population for patients who would likely benefit from a statin now or in the near future but are not currently prescribed a statin. Patients will be eligible to be considered for enrollment if they 1) are aged 40-75 years; 2) have no history of statin use; 3) have received VA care for at least the prior 6 months; 4) are a patient of an enrolled provider, and 5) meet at least ONE of the following criteria:

a. Have CVD (determined from ICD codes)
b. Have diabetes
c. Have an LDL cholesterol value ≥190 mg/dL
d. Have a 10-year CVD risk of ≥7.5%, calculated with the ACC/AHA 2013 pooled risk equations

At the study's baseline and then as needed during the enrollment period, the study team will perform a database query to identify all patients at VA Boston meeting the above eligibility criteria. The resulting list of eligible patients will be stored as a database on an encrypted VINCI server. To increase the enrollment of female participants, non-Veteran female patients, such as spouses of Veterans, who receive VA primary care services and who meet the above eligibility criteria are eligible to participate.

iii. Patient consent: As providers consent to participate in the study, the study staff will send out informed consent letters to their potentially eligible patients at VA Boston, giving the patients the opportunity to opt in to study participation. This patient informed consent letter (included with this submission) will describe the study

in detail, including *SLCO1B1* testing and its interpretation, benefits and risks to the patient, confidentiality, and data security. In response to this letter, patients will call a member of the study team to ask questions about the study, opt in to participation, or decline participation (see Patient Opt-in Phone Script included with this submission). If a patient declines participation, their name will be removed from the list of eligible patients for potential enrollment. If a patient opts in to participation, their name will be added to the list of consented patients. If a patient does not call the study staff, the study staff may call the patient to confirm that they received the informed consent letter and to ask whether they would like to opt in or out of study participation (see Patient Outreach Phone Script included with this submission). Patients may withdraw their consent at any time by calling the study staff at the phone number listed on their mailed informed consent letter. Patient consent does not constitute patient enrollment; consented patients can only be enrolled in the study if their provider then chooses to sign an order for *SLCO1B1* testing for them, as described below. Patient consent will be tracked by study staff and will include logging the date opt-in letters are mailed and when phone consent is obtained. If an enrolled provider wants to refer a patient directly to the study staff for recruitment, he/she may do so by contacting the study staff (by phone or by encrypted email) and providing the patient's name and last 4 SSN. The eligibility of any referred patient will be verified in CPRS, and the study staff will contact the patient to obtain informed consent.

iv. Patient enrollment and randomization: Although patient consent occurs at the study's baseline, patient enrollment occurs in relative real-time thereafter, to take advantage of eligible clinical blood samples as they are ordered during routine clinical care. Each night during the enrollment period, an informatics-based algorithm will perform a database query to identify any eligible patients who have electronic orders for any testing in an EDTA tube (e.g. a complete blood count, CBC, or a hemoglobin A1c) at the VA in the prior 3 days (generally the day prior, but up to 3 days to account for weekends). The next day, a member of the study staff will review this list of eligible patients with eligible lab orders and will cross-reference it against the list of consented patients, making note of any non-consented patients to not include in study enrollment. For any eligible, consented patient, the study staff will create an order for *SLCO1B1* testing in CPRS, to be forwarded to the patient's provider for signature. The provider's signature of the lab order enrolls the patient in the study, provided the blood sample is adequate for *SLCO1B1* genotyping. If the provider discontinues the order or does not sign the order within the timeframe that the laboratory saves clinical samples (generally about 7 days), the patient will not be enrolled. If needed, the study staff can cap the number of eligible patients that a given provider will be presented per week, to minimize the number of CPRS view alerts and reduce provider burden. The study staff will randomize enrolled patients to the PGx+ vs. PGx- groups. If a consented patient tells the study staff that he/she is particularly interested in having the *SLCO1B1* test ordered, the study staff may convey this information to the patient's provider by encrypted email and create a lab order for the provider to considering signing. This option would not be routinely suggested by study staff.

A total of 408 patients will be enrolled in this RCT.

v. Laboratory testing and reporting: Once an eligible blood sample is collected and the provider has signed the *SLCO1B1* order, study staff will contact the VA laboratory to ensure that the sample is adequate for *SLCO1B1* genotyping. If it is, the patient will be enrolled in the study, the study staff will randomize the patient to the PGx+ vs. PGx- group, and the staff will notify the VA laboratory to send the patient's clinical blood sample for *SLCO1B1* testing. The VA Boston laboratory will use standard clinical workflow to send out labeled, identified samples, using a common carrier delivery service and chain of custody, to process and send these samples to Boston Heart Diagnostics (BHD) in Framingham, MA, for *SLCO1B1* rs4149056 genotyping. The Boston Heart Diagnostics laboratory will store all blood samples received from the VA laboratory in a freezer. After a blood sample is tested and the result is reported back to the VA Boston laboratory, Boston Heart Diagnostics will destroy the sample, generally within 7 days and not to exceed 60 days. No research data will be sent outside of the VA. All samples (PGx+ and PGx-) will be genotyped only for rs4149056 in *SLCO1B1* using the BHD

CLIA-certified and CAP-accredited PCR assay. BHD will return *SLCO1B1* results for both arms to study staff using secure fax. Study staff will store these results in a data file on a secure VINCI server. Using encrypted VA email, study staff will send each *SLCO1B1* result to the VA clinical laboratory staff to be reported in CPRS according to the patient's random assignment (PGx+ vs. PGx-). Results for patients randomized to the PGx+ arm (immediate reporting) will be distributed to VA clinical laboratory staff for immediate posting to CPRS. Results for patients randomized to the PGx- arm (delayed reporting) will be distributed to VA clinical laboratory staff for posting to CPRS after the patient has completed (or declines to complete) the 12-month phone survey described below. If the patient cannot be reached for the 12-month phone survey after at least 3 attempts, the results will be distributed to the VA clinical laboratory staff for posting in CPRS, no later than 15 months from the patient's date of enrollment. Each *SLCO1B1* genotype result will appear as a view alert for the ordering provider. The PI or member of the study team may also send the provider an encrypted email alerting them that the results have been reported. The CPRS results screen will include the following information:

SLCO1B1 genotype	T/T or T/C or C/C
Transporter Function	Normal, Decreased, or Poor Function
Simvastatin myopathy risk	T/T – Typical<br>T/C - Increased<br>C/C – Markedly increased
Interpretation	T/T – Individuals with the T/T genotype have normal ability to metabolize statins. Standard statin dosing, if indicated, is recommended.<br><br>T/C – Individuals with the T/C genotype have decreased ability to metabolize statins and have a 4-fold increased risk of simvastatin-related myopathy. Simvastatin at a dose of ≤20 mg or an alternate statin, if indicated, is recommended.<br><br>C/C – Individuals with the C/C genotype have markedly decreased ability to metabolize statins and have a 17-fold increased risk of simvastatin-related myopathy. Simvastatin at a dose of ≤20 mg or an alternate statin, if indicated, is recommended.

vi. <u>Observation period and outcomes</u>: Providers will act on the *SLCO1B1* results according to their judgment, as they would in routine clinical care. Such actions might include sending a patient letter, calling the patient, scheduling a follow-up appointment, and/or discussing therapeutic options with the patient, including lifestyle modification and/or pharmacotherapy. The study intervention protocol ends at the point when the study staff deliver the *SLCO1B1* results to the provider, but providers have a *SLCO1B1* lab results letter template available to them in CPRS that they may edit and use if they see fit (see attached "Vassy 2993 CPRS Patient Results Letter Template"). Twelve months after enrollment, the study staff will query the Corporate Data Warehouse (CDW) for study outcomes, including statin prescriptions, laboratory values (e.g., LDL cholesterol), and documentation of statin side effects.

vii. <u>End-of-study survey and results letters</u>: Twelve months after a patient's enrollment date, study staff will call the patient to administer a brief, 3-minute telephone survey about medications, side effects, and recall of genetic test results (see attached "Vassy 2993 I-PICC 12-month survey"). After this survey, patients in both arms (PGx+ and PGx-) will be mailed a letter with their study results (see attached "Vassy 2993 I-PICC 12-month

letter”). A copy of this letter will also be sent by encrypted email to the patient’s provider. Patients may decline to complete the phone survey and still receive their study results. For patients not responsive to initial outreach, study staff will attempt to administer the 12-month end-of-study survey up to and no later than 15 months from the patient’s initial date of enrollment. Pharmacogenetic test results for the PGx- arm will be reported to patients, providers, and CPRS only after a patient’s completion or affirmed decline of this survey. If a patient has not responded to 12-month end-of-study survey attempts for a period of 3 months (or 15 months from his or her initial enrollment date), study staff will distribute a 12-month letter (see attached “Vassy 2993 I-PICC 12-month letter”) to the patient and report results to his or her provider and CPRS (PGx-). After the 12-month observation period is complete for the last enrolled patient, an “order check” will be programmed in the Boston instance of CPRS. Similar to a drug allergy alert, this order check will trigger a pop-up alert window if any provider at VA Boston orders simvastatin on one of the approximately 80 patients anticipated to have at least one copy of the C risk allele. Providers may override this alert if they wish.

(d) Data collection: Using the procedures described above, the following data will be collected during the study.

i. Provider characteristics: Demographic information about participating providers will be obtained from the provider data files at VA Boston.

ii. Patient eligibility: The following data will be collected from the CDW to determine the list of eligible patients to whom a patient informed consent letter may be mailed:

1. Providers (to determine whether patients sees an eligible, enrolled provider)
2. Date of birth (to determine age)
3. Prior and current medication prescriptions (to determine history of statin use and treatment for hypertension)
4. TIU notes (to perform text search to confirm absence of prior statin treatment)
5. ICD codes and problem list (to determine history of CVD and diabetes)
6. Laboratory results, race, smoking history, and blood pressure (to calculate 10-year CVD risk by ACC/AHA equations)
7. Dates of VA encounters (to determine whether patient has received VA care for ≥6 months)

iii. Patient enrollment: The study staff will perform nightly data queries of the EHR data systems to determine which eligible, consented patients have had an eligible lab order in the prior 1-3 days (a whole-blood sample, such as CBC or hemoglobin A1c).

iv. *SLCO1B1* results: The study staff will receive *SLCO1B1* results from Boston Heart Diagnostics through secure fax. Using encrypted email, study staff will send *SLCO1B1* results to the VA laboratory immediately (PGx+) or as early as 12 months, but no later than 15 months (pending 12-month end-of-study survey completion) after a patient’s enrollment (PGx-) to be reported in CPRS.

v. Outcomes from VINCI/CDW: For each enrolled patient and a cohort of matched unenrolled control patients, the study staff will obtain the following data from the CDW during the 12 months before and 12 months after enrollment:

1. Outpatient and inpatient encounters: station/clinic, date, provider
2. ICD codes and problem list and associated dates
3. Pharmacy data: all medications, doses, and dates of prescriptions, fills, renewals, and refills
4. Medication allergies: medication, reaction, date, and provider

5. All laboratory values and dates, including LDL cholesterol, creatinine kinase (CK) values, and liver enzymes
6. All TIU notes
7. Data on healthcare costs from economic datasets, such as the Health Economics Resource Center (HERC) cost datasets and Managerial Cost Accounting (MCA) datasets.

vi. Outcomes from 12-month survey: Study staff will administer a telephone survey to enrolled patients to collect the following data:

1. Use of and side effects from statin and other cholesterol-lowering medications in the prior 12 months
2. Brief adaptation of beliefs about medications questionnaire
3. Recall of genetic test results

(e) Statistical analysis

Safety: Our primary safety outcome will be concordance with CPIC guidelines for safe simvastatin use 12 months after enrollment. For CPIC concordance, we will compare each enrolled patient's *SLCO1B1* genotype and statin type and dose at the end of the 12-month observation period to the CPIC guidelines for safe simvastatin prescribing:

T/T genotype: Standard simvastatin prescribing
C/T or C/C genotype: Avoid 40 mg simvastatin; consider 20 mg simvastatin or alternate statin

We will consider potentially unsafe simvastatin dosing to include 80 mg daily for any person and 40 mg daily for any person with a CT or CC genotype. We will consider all other combinations potentially safe, including no simvastatin prescription or use of a statin other than simvastatin. This will generate a 2-level safety outcome (potentially safe vs. potentially unsafe simvastatin prescription) for each participant. The secondary safety outcome will be one-year incidence of statin myopathy, determined primarily by chart review of all 408 patients but also by the NLP algorithm under development in IRB #2953 ("Clinical Safety & Efficacy of Pharmacogenetics in Veteran Care").

CVD prevention: Our primary outcome for appropriate CVD prevention will be LDL levels 12 months after enrollment. The secondary outcome for CVD prevention will be concordance at 12 months with ACC/AHA guidelines for statin use in CVD prevention, which recommend statins of specific intensities (moderate or high) for distinct patient populations (see Table)[9]. Using patient characteristics and prescription data, we will generate a 2-level CVD prevention outcome (concordant vs. non-concordant) for each participant, a measure of whether the Veteran's statin prescription is adequate for his/her level of CVD risk.

**High-Intensity**	**Moderate-Intensity**	**Low-Intensity**
Atorvastatin 40-80 mg<br>Rosuvastatin 20-40 mg<br>Simvastatin 80 mg*	Atorvastatin 10-20 mg<br>Rosuvastatin 5-10 mg<br>Simvastatin 20-40 mg<br>Pravastatin 40-80 mg<br>Lovastatin 40 mg<br>Fluvastatin XL 80 mg<br>Fluvastatin 40 mg bid<br>Pitavastatin 2-4 mg	Simvastatin 10 mg<br>Pravastatin 10-20 mg<br>Lovastatin 20 mg<br>Fluvastatin 20-40 mg<br>Pitavastatin 1 mg
**Recommended For:**	**Recommended For:**	
1. Clinical CVD, age ≤75 y<br>2. LDL ≥ 190mg/dL<br>3a. Diabetes, age 40-75 y, 10-y CVD risk ≥7.5%	3b. Diabetes, age 40-75 y, 10-y CVD risk <7.5%<br>4. 10-y CVD risk ≥7.5%, age 40-75 y	

Analysis plan: For the primary safety outcome, we will use generalized estimating equations with a logit link function, accounting for clustering by provider, to test the null hypothesis that the proportion of patients whose prescriptions meet CPIC guidelines on the 365th day after enrollment, *p*, does not differ between the PGx+ and PGx- arms (superiority design). Stated formally: H_0: $p_1=p_2$; H_a: $p_1 \neq p_2$, where p_1 is the proportion meeting CPIC guidelines in the PGx+ group and p_2 is the proportion meeting CPIC guidelines in the PGx- group, as assessed one year after enrollment. The primary CVD prevention outcome is change in LDL, ∂, defined as the baseline LDL level subtracted from the most recent LDL value prior to or on the 365th day after the baseline LDL. We will use generalized estimating equations with an identity link function, accounting for clustering by physician, to test the null hypothesis that ∂ will be greater in the PGx- arm than in the PGx- arm by 6% one year after enrollment (non-inferiority design). We will also perform generalized estimating equations with a logit link function, accounting for clustering by ordering physician, to test the between-group differences for the secondary outcomes of ACC/AHA concordance and proportion with statin-related myopathy after 12 months.

Understanding the financial impact of *SLCO1B1* testing is a critical element for understanding its clinical utility. Similar to Dr. Vassy's prior work in the MedSeq Project trial of genome sequencing[52], we will also undertake a cost analysis alongside the I-PICC Study randomized controlled trial, using guidelines published by the International Society for Pharmacoeconomics and Outcomes Research and the Second Panel on Cost-Effectiveness in Health and Medicine[53,54]. Briefly, to understand the impact of *SLCO1B1* testing on follow-up healthcare costs, we will assess costs over the 12 months following enrollment for I-PICC Study patient-participants. We will use a microcosting approach[55] to estimate the costs of *SLCO1B1* testing itself and will use cost data from the HERC and MCA datasets to determine patient-level healthcare costs in the 12 months after enrollment. We will use multivariable linear regression to compare arithmetic mean costs between the 2 randomization arms, as recommended[53].

Power calculation: With 408 patients for the RCT, we have 80% power at a 2-sided α=0.05 to detect a difference of at least 15% (superiority design) in our primary safety outcome: the proportions meeting CPIC guidelines in the 2 arms. This sample size assumes a design effect of 1.36 to account for clustering by physician (cluster size of 10 patients/physician and an intracluster correlation of 0.04)[56] and assumes that concordance with CPIC guidelines in the PGx- arm ranges from 60-100% (or, equivalently, 0-40%)[57]. For our primary CVD prevention outcome, change in LDL, ∂, a sample size of 408 patients gives >80% power to state that the upper limit of a 1-sided 95% confidence interval excludes a difference of >6% favoring the PGx- arm (non-inferiority design), assuming a common standard deviation of ∂ in the two main arms of 20% and a design effect of 1.36[58].

Proportion meeting CPIC guidelines in PGx- arm	50%	60%	70%	80%	85%
Sample size (total in both arms)	334	300	236	146	90
Sample size after design effect of 1.36	455	408	321	199	122

Sample size calculations primary safety outcome. Data are total sample sizes required for 80% power at a 2-sided α=0.05 to detect a difference of at least 15% in the concordance with CPIC guidelines between the PGx+ and PGx- arms.

(f) Potential limitations

i. Low enrollment due to fewer eligible patients than expected or eligible patients who do not consent: If necessary, it will be straightforward to take advantage of the Clinical Trials Network to expand this trial beyond VA Boston to other primary care sites within VISN 1, an integrated system of 8 medical centers and 35 outpatient clinics across New England, employing 316 primary care practitioners and treating >240,000 patients annually at 2.5 million outpatient visits.

ii. Few cases of statin initiation: Our choices of outcomes (concordance with CPIC and ACC/AHA guidelines and LDL cholesterol levels) do not require the majority of patients to be initiated on statins during the

observation period to achieve important results. However, we have chosen the patient eligibility criteria to enrich the population for Veterans who are likely to benefit from statin therapy according to current guidelines.

iii. Logistical difficulties in sample send-out to the BHD laboratory and delivery of *SLCO1B1* results to providers: An alternative plan would be to send our samples for genotyping at the CLIA-certified and CAP-accredited Partners Laboratory for Molecular Medicine, under the directorship of Dr. Heidi Rehm, a collaborator with Dr. Vassy on the MedSeq Project.

(h) **Human Studies Section:**

(1) **Risk to Subjects**

(a) Human Subjects Involvement and Characteristics

i. Providers: We will recruit all interested providers in Primary Care or Women's Health at VA Boston to participate in the study, regardless of age, gender, or years of experience.

ii. Patients: Patients will be eligible to enroll in this trial if they 1) are aged 40-75 years; 2) have no history of statin use; 3) have received VA care for at least the prior 6 months; 4) are a patient of an enrolled provider, and 5) meet at least ONE of the following criteria:

a. Have CVD (determined by ICD codes)
b. Have diabetes
c. Have an LDL cholesterol value ≥190 mg/dL
d. Have a 10-year CVD risk of ≥7.5%, calculated with the ACC/AHA pooled risk equations

We have chosen these eligibility criteria to enrich the study population with established primary care patients whose providers may initiate statin therapy in the next year. The study sample will likely reflect the overall composition of the VA patient population, although we will include the Women's Health clinic at the VA to increase the representation of women. Additionally, non-Veteran patients who meet the above eligibility criteria will also be eligible, for the purpose of increasing female enrollment. Elderly patients up to age 75 will be eligible to participate. Pregnant women, children, prisoners, and institutionalized individuals will not be included in the study.

(b) Sources of Materials

Demographic information about the participating providers will be obtained from the provider data files at VA Boston. We will collect patient data from existing VA Boston clinical information systems: CPRS and the VA pharmacy data. Data will include patient demographics, medical conditions, CVD risk factors, prescriptions, laboratory values including LDL cholesterol levels, and TIU notes. *SLCO1B1* genotype will be obtained from PCR testing performed on existing patient blood samples obtained as a part of routine clinical care (i.e., CBC or hemoglobin A1c testing). A 12-month telephone survey will collect data about patient medications, side effects, and recall of genetic test results. Study staff will enter patients' verbal responses into a study database behind the VA firewall.

(c) Potential Risks

i. Risks to providers: VA providers are considered a vulnerable population. The risks to providers participating in this study are minimal. These include the risk of malpractice litigation if a provider is perceived to have inappropriately under-dosed a statin and a patient goes on to have a CVD event; if the physician's statin

prescribing practices are thought to have resulted in statin-related myopathy. These risks are not dissimilar to those of current standard of care around statin use, CVD prevention, and the management of hereditary conditions. Drs. Jacqueline Spencer and Megan Gerber, Directors and Primary & Ambulatory Care and Women's Health, respectively, have endorsed the design of the trial of statin pharmacogenetics testing as presenting minimal burden to providers (see attached letter of support). There is also a risk of breach of data privacy; however, this study will not be collecting protected health information from providers as research subjects. For the trial of statin pharmacogenetics testing, provider informed consent will be documented in the medical records of fake patients in CPRS, accessible only to study staff, and a paper copy of the CPRS consent note will be printed, labeled and stored in the research records of the enrolled providers. Providers who prefer to mail signed informed consent documents to the study team will have their consent forms labeled and securely stored in their research records. All provider consent documents will be filed in a locked cabinet located at the Jamaica Plain Campus of VA Boston, Building 9, Room 425C, accessible only to study staff.

ii. Risks to patients: This trial of statin pharmacogenetics testing poses minimal risk to patients. The potential risks are that providers may be more reluctant to start appropriate statin therapy for patients with elevated CVD risk and/or patients may be more reluctant to adhere to prescribed statin therapy. However, in routine clinical care, there is already much variation in provider behavior around statin prescribing and patient behavior around medication adherence. Thus, this study poses risks not dissimilar to those of current standard of care around statin use and CVD prevention. There is no physical risk to enrolled patients as a patient's enrollment into the study occurs after a blood draw has been performed as part of clinical care. Participation will incur no costs for patients and will have no effect on patients' clinical care or healthcare benefits. The risk of breach of data privacy will be minimized using the data security measures described below.

(2) **Adequacy of Protection from Risks**

(a) Recruitment and Informed Consent

i. Providers: Providers will be educated about this study by the study staff (principal investigator, project manager, and/or research assistant) through presentations at VA Boston Primary Care and Women's Health staff meetings and through individual e-mail outreach. Informational presentations and flyers (see attached) will include a description of the association between *SLCO1B1* genotype and simvastatin-related myopathy and the CPIC guidelines for simvastatin prescribing according to *SLCO1B1* genotype. These materials will also briefly describe the study protocol, including study workflow and patient enrollment. After these educational materials have been presented and distributed, an email will be sent to providers instructing them how to give informed consent if they would like to participate in the study (see attached Initial Provider Recruitment email). Providers have two options for how to give informed consent: through CPRS or on a paper-based form. A 'testpatient' view alert from a fake patient's CPRS record will be sent to each eligible provider. This 'testpatient' view alert will accompany a progress note containing the text of the provider informed consent (see attached), which will contain the elements of informed consent and detail the study procedures. By signing this 'testpatient' note, the provider is agreeing to enroll in the study as a research subject and allow the study team to contact his/her eligible patients. This approach for obtaining informed consent from providers was adopted from the Diuretic Comparison Project (DCP) (CSP #597) study protocol that has been approved by the VA Central IRB. For providers who would prefer to sign an informed consent form outside of CPRS, the recruitment email from the study staff will also include the informed consent form as a pdf document, which the provider may print, sign, and return to the study staff by intercampus mail. If a provider does not sign the CPRS order or return a paper copy of the informed consent form within a week, the study staff may send a follow-up email (see attached Follow-up Provider Recruitment email), reminding them about the opportunity to enroll in the study by signing the CPRS alert or returning a signed paper copy of the informed consent form. Provider consent will be tracked by study staff in a secured data file that includes all eligible providers at VA Boston. If and when a provider

consents to participate, the study staff will log that he/she is “CONSENTED” and the date and method of consent (CPRS or paper copy). Providers who expressly notify the staff that they are not interested in participating will be designated as “OPTOUT” in this file. All other providers will remain designated as “ELIGIBLE” in the file. Only CONSENTED providers will be considered enrolled in the study, and only the patients of consented providers will be eligible for patient enrollment. After enrolling in this study, a provider may unenroll at any time by contacting the Principal Investigator or study coordinator by phone and request that they no longer receive orders to enroll eligible patients. These providers will be designated as “UNENROLLED” in the provider file.

ii. Patients: As providers consent to participate in the study, the study staff will send out informed consent letters to their potentially eligible patients at VA Boston, giving the patients the opportunity to opt in to study participation. This patient informed consent letter (included with this submission) will describe the study in detail, including *SLCO1B1* testing and its interpretation, benefits and risks to the patient, confidentiality, and data security. In response to this letter, patients will call the study coordinator to ask questions about the study, opt out of participation, or opt in to participation (see Patient Opt-in Phone Script included with this submission). If a patient opts out of participation, their name will be removed from the list of eligible patients for potential enrollment. If a patient opts in to participation, their name will be added to the list of consented patients. If a patient does not call the study staff, the study staff may call the patient to confirm that they received the informed consent letter and to ask whether they would like to opt in or out of study participation (see Patient Outreach Phone Script included with this submission). Patients may withdraw their consent at any time by calling the study staff. Patient consent does not constitute patient enrollment; consented patients can only be enrolled in the study if their provider then chooses to sign an order for *SLCO1B1* testing for them. Patient consent will be tracked by study staff and will include logging the date opt-in letters are mailed and when phone consent is obtained. If an enrolled provider wants to refer a patient directly to the study staff for recruitment, he/she may do so by contacting the study staff (by phone or by encrypted email) and providing the patient’s name and last 4 SSN. The eligibility of any referred patient will be verified in CPRS, and the study staff will contact the patient to obtain informed consent.

Patient consent occurs at the study’s baseline, but patient enrollment occurs in relative real-time thereafter, to take advantage of eligible clinical blood samples as they are ordered during routine clinical care. Each night during the enrollment period, an informatics-based algorithm will perform a database query to identify any eligible patients who with electronic orders for any testing in an EDTA tube (e.g. a complete blood count, CBC, or a hemoglobin A1c) at the VA in the prior 3 days (generally the day prior, but up to 3 days to account for weekends). The next day, a member of the study staff will review this list of eligible patients who have eligible lab orders and will cross-reference it against the list of consented patients, making note of any non-consented patients to not include in study enrollment. For any eligible, consented patient, the study staff will create an order for *SLCO1B1* testing in CPRS, to be forwarded to the patient’s provider for signature. The provider’s signature of the lab order enrolls the patient in the study, provided the sample is adequate for *SLCO1B1* testing. If the provider has not signed the order, the study staff may contact the provider by encrypted e-mail, asking him/her to consider signing the order if deemed appropriate. If the provider discontinues the order or does not sign the order within the timeframe that the laboratory saves clinical samples (generally about 7 days), the patient will not be enrolled. If a consented patient tells the study staff that he/she is particularly interested in having the *SLCO1B1* test ordered, the study staff may convey this information to the patient’s provider by encrypted email and create a lab order for the provider to considering signing. This option would not be routinely suggested by study staff. Given the opt-in mechanism of obtaining informed patient consent and the fact that only a fraction of consented patients will ultimately be enrolled in the study, we are requesting a waiver of the requirement to document informed patient consent (see attached memo).

At the end of the study (12 months after enrollment), study staff will call each enrolled patient for a brief telephone survey, first obtaining verbal consent to do so (see attached "Vassy 2993 I-PICC 12-month survey"). Attempts to administer the 12-month end-of-study survey to enrolled patients will not exceed 15 months from their initial dates of enrollment.

(b) Protection Against Risk: The risks to participating providers and patients are minimal and not dissimilar from routine clinical care, where there is already much variation in provider behavior around statin prescribing and patient behavior around medication adherence. The misinterpretation and misuse of *SLCO1B1* genotype results will be minimized through the clear, concise, and evidence-based test interpretation and recommendation provided with each genotype result.

i. Adverse events: An adverse event (AE) will be defined as any unanticipated or unintended medical occurrence or worsening of a sign or symptom (including an abnormal laboratory finding) or disease in a study subject, which does not necessarily have a causal relationship with the study condition, procedure(s) or study agent(s), that occurs after the informed consent is obtained. Pre-existing conditions or illnesses which are expected to exacerbate or worsen are not considered adverse events and will be accounted for in the subject's medical history. A serious adverse event (SAE) will be defined as an AE resulting in one of the following outcomes: death during the 12 months after enrollment, life threatening event (defined as an event that places a participant at immediate risk of death), inpatient hospitalization, and any other condition which, in the judgment of the investigator, represents a significant hazard, such as an important medical event that does not result in one of the above outcomes. An event may be considered an SAE when it jeopardizes the participant or requires medical or surgical intervention to prevent one of the outcomes listed above. AEs may be observed by the study staff or volunteered by VA providers and patients. All AEs or SAEs will be assessed for relationship to the study research procedures, to determine whether study participation was likely to have caused the AE/SAE. AEs related to study participation that are reported to research personnel will be recorded on an AE form in an electronic database. All deaths and study-related AEs will be reported to the IRB in keeping with VA protocols.

ii. Data security: Risk of breach of confidentiality will be minimized through the appropriate management and security of clinical data per VA and HIPAA protocols for use of research data. Patient protected health information (PHI) will be delinked from the final analytic dataset; no provider PHI will be collected or stored. All data will be retained within the VA. Data will be securely transmitted using VA approved methods. We will use FIPS 140-2 validated encryption. Patient and provider data files (source and analytic) will be stored behind the VA firewall, on a drive created by VINCI specifically to house the data for this research project. A copy of patient mailing data only will be downloaded outside of VINCI in a VA secured, study specific SharePoint site, and behind the VA firewall where strict permissions will be set to limit viewing to IRB approved study personnel. This will be done to allow for the use of the Microsoft mail merge software so patient letters and address labels can be created and printed in batches, increasing patient enrollment numbers to meet the study's grant time table. Patient mailing data will be in the form of CSV files and may include identifying variables for both patients and providers. Variables for patients/providers may include: ID, full name, title, institution code/ID, gender, mailing address and any associated flags (i.e. temporary address), patient-provider relationship information, or other similar variables that are required to be able to send mail or that are named in the IRB-approved patient letter template. The use of the mail merge system can be completed within the secure SharePoint environment. Only study personnel credentialed and approved by the IRB and VA Research & Development committees will have access to study data in both the VINCI and SharePoint environments. Software to be used in this study, including SQL Server Management Studio and R, is already in place on VINCI servers, and no additional licenses will be required. Once study team members are no longer a part of the research team, their access to data and research materials will be terminated in both VINCI and VA secured SharePoint. No outsider can have access to any of these files. Since the data used will be aggregate, they cannot be used to uniquely identify any patient. We will not allow

any unauthorized access to our servers or our datasets. No PHI (scrambled SSNs or dates) will be released to the public, nor will they be published in any medical journal. Mobile devices will not be used for data collection or management. Suspected information security and privacy incidents will be reported within one hour to the Information Security and Privacy Officers and Research Administration. Data will be kept indefinitely or until the law allows their destruction in accordance with the VA Record Control Schedule. Electronic records will be destroyed, when allowed, in a manner in which they cannot be retrieved.

A research data repository will be created from the trial data. The data repository will be housed in data files on a secure VINCI server, password-protected and accessible only to IRB-approved personnel. Data will be securely transmitted using VA approved methods. We will use FIPS 140-2 validated encryption. The data in the repository will not include identifiers except for the study ID. A data file linking the study ID to patient identifiers (e.g. Social Security number) will be stored in a separate location on a secure VINCI server, enabling investigators to access the data repository but not the ID linker file unless they also have IRB-approved access to do so. This data repository will allow IRB-approved investigators, both inside and outside VA, to analyze de-identified genome sequence data. Interested investigators will need to obtain IRB approval and complete a Combined Data Use-Data Transfer Agreement. The principal investigator or another member of the study team will keep the names and contact information of individuals approved to access the data repository in a data file, along with the dates that approved access expires. The data file linking patient identifiers (e.g. SSN) to the study ID will be provided to investigators only with IRB approval to do so.

(3) **Potential Benefit of the Proposed Research to the Subject and Others.**

The benefits to providers and patients participating in this study include the potential for them to engage with each other in therapeutic conversations about the risks and benefits of statin therapy. Learning a patient's *SLCO1B1* genotype may help physicians and patients reduce the risk of statin myopathy, and patients may be more adherent to therapy they feel is personalized to them. Society will also benefit from the knowledge to be learned about the impact of introducing *SLCO1B1* testing into clinical care. These potential benefits outweigh the minimal potential risks to providers and patient

(4) **Importance of the Knowledge to be Gained.**

There is an increasing eagerness among scientists, clinicians, patients, and health systems to introduce pharmacogenetic information, including *SLCO1B1* genotype, into clinical care. However, it is unknown how this will impact clinical outcomes. This study will evaluate the impact that *SLCO1B1* genotype testing has on statin use, LDL cholesterol levels, and risk of statin myopathy within a health system while posing no more than minimal harm to the providers and patients in that system.

(5) **Resources**

(a) **Research Space**:

Enrolled patients' blood samples will be sent for *SLCO1B1* genotyping to the Boston Heart Diagnostics laboratory in Framingham, MA.

(b) **Other Research Resources:**

The research will be conducted in VA Boston research space in the Section of General Internal Medicine and the Massachusetts Veterans Epidemiology Research and Information Center (MAVERIC), equipped with computers networked to the VA intranet for access to VINCI servers.

(i) **Publications from Last Funding Period (as applicable)**: N/A

(j) **Literature Citations (as applicable)**.

1. Martin SS, Blumenthal RS. Concepts and Controversies: The 2013 American College of Cardiology/American Heart Association Risk Assessment and Cholesterol Treatment Guidelines. *Ann. Intern. Med.* Jan 28 2014.

2. Kochanek K, Xu J, Murphy S, Miniño A, Kung H. Deaths: final data for 2009. *National Vital Statistics Report.* 2011;60(3).

3. Kazis LE, Ren XS, Lee A, et al. Health status in VA patients: results from the Veterans Health Study. *Am. J. Med. Qual.* Jan-Feb 1999;14(1):28-38.

4. Agha Z, Lofgren RP, VanRuiswyk JV, Layde PM. Are patients at Veterans Affairs medical centers sicker? A comparative analysis of health status and medical resource use. *Arch. Intern. Med.* Nov 27 2000;160(21):3252-3257.

5. Wilson PW, D'Agostino RB, Levy D, Belanger AM, Silbershatz H, Kannel WB. Prediction of coronary heart disease using risk factor categories. *Circulation.* May 12 1998;97(18):1837-1847.

6. National Heart Lung and Blood Institute. *Third Report of the National Cholesterol Education Program (NCEP) Expert Panel on Detection, Evaluation, and Treatment of High Blood Cholesterol in Adults (Adult Treatment Panel III).* National Institutes of Health; September 2002 2002. NIH Publication Number 02-5215.

7. Mills EJ, Rachlis B, Wu P, Devereaux PJ, Arora P, Perri D. Primary prevention of cardiovascular mortality and events with statin treatments: a network meta-analysis involving more than 65,000 patients. *J. Am. Coll. Cardiol.* Nov 25 2008;52(22):1769-1781.

8. Baigent C, Keech A, Kearney PM, et al. Efficacy and safety of cholesterol-lowering treatment: prospective meta-analysis of data from 90,056 participants in 14 randomised trials of statins. *Lancet.* Oct 8 2005;366(9493):1267-1278.

9. Stone NJ, Robinson J, Lichtenstein AH, et al. 2013 ACC/AHA Guideline on the Treatment of Blood Cholesterol to Reduce Atherosclerotic Cardiovascular Risk in Adults: A Report of the American College of Cardiology/American Heart Association Task Force on Practice Guidelines. *Circulation.* Nov 12 2013.

10. Ho PM, Magid DJ, Shetterly SM, et al. Medication nonadherence is associated with a broad range of adverse outcomes in patients with coronary artery disease. *Am. Heart J.* Apr 2008;155(4):772-779.

11. Zhang H, Plutzky J, Skentzos S, et al. Discontinuation of statins in routine care settings: a cohort study. *Ann. Intern. Med.* Apr 2 2013;158(7):526-534.

12. Newby LK, LaPointe NM, Chen AY, et al. Long-term adherence to evidence-based secondary prevention therapies in coronary artery disease. *Circulation.* Jan 17 2006;113(2):203-212.

13. Ellis JJ, Erickson SR, Stevenson JG, Bernstein SJ, Stiles RA, Fendrick AM. Suboptimal statin adherence and discontinuation in primary and secondary prevention populations. *J. Gen. Intern. Med.* Jun 2004;19(6):638-645.

14. Watanabe JH, Bounthavong M, Chen T, Ney JP. Association of Polypharmacy and Statin New-User Adherence in a Veterans Health Administration Population A Retrospective Cohort Study. *Ann. Pharmacother.* 2013;47(10):1253-1259.

15. Shalev V, Chodick G, Silber H, Kokia E, Jan J, Heymann AD. Continuation of statin treatment and all-cause mortality: a population-based cohort study. *Arch. Intern. Med.* Feb 9 2009;169(3):260-268.

16. Rasmussen JN, Chong A, Alter DA. Relationship between adherence to evidence-based pharmacotherapy and long-term mortality after acute myocardial infarction. *JAMA.* Jan 10 2007;297(2):177-186.

17. Silva MA, Swanson AC, Gandhi PJ, Tataronis GR. Statin-related adverse events: a meta-analysis. *Clin. Ther.* Jan 2006;28(1):26-35.
18. Law M, Rudnicka AR. Statin safety: a systematic review. *Am. J. Cardiol.* Apr 17 2006;97(8A):52C-60C.
19. Armitage J. The safety of statins in clinical practice. *Lancet.* Nov 24 2007;370(9601):1781-1790.
20. Wilke RA, Ramsey LB, Johnson SG, et al. The clinical pharmacogenomics implementation consortium: CPIC guideline for SLCO1B1 and simvastatin-induced myopathy. *Clin. Pharmacol. Ther.* Jul 2012;92(1):112-117.
21. Thompson PD, Clarkson PM, Rosenson RS. An assessment of statin safety by muscle experts. *Am. J. Cardiol.* Apr 17 2006;97(8A):69C-76C.
22. Buettner C, Davis RB, Leveille SG, Mittleman MA, Mukamal KJ. Prevalence of musculoskeletal pain and statin use. *J. Gen. Intern. Med.* Aug 2008;23(8):1182-1186.
23. Kashani A, Phillips CO, Foody JM, et al. Risks associated with statin therapy: a systematic overview of randomized clinical trials. *Circulation.* Dec 19 2006;114(25):2788-2797.
24. Joy TR, Monjed A, Zou GY, Hegele RA, McDonald CG, Mahon JL. N-of-1 (single-patient) trials for statin-related myalgia. *Ann. Intern. Med.* 2014;160(5):301-310.
25. Tompkins R, Schwartzbard A, Gianos E, Fisher E, Weintraub H. A current approach to statin intolerance. *Clin. Pharmacol. Ther.* Apr 11 2014.
26. Ahmad Z. Statin intolerance. *Am. J. Cardiol.* May 15 2014;113(10):1765-1771.
27. Link E, Parish S, Armitage J, et al. SLCO1B1 variants and statin-induced myopathy--a genomewide study. *N. Engl. J. Med.* Aug 21 2008;359(8):789-799.
28. Hagenbuch B, Meier PJ. Organic anion transporting polypeptides of the OATP/ SLC21 family: phylogenetic classification as OATP/ SLCO superfamily, new nomenclature and molecular/functional properties. *Pflugers Arch.* Feb 2004;447(5):653-665.
29. Konig J, Seithel A, Gradhand U, Fromm MF. Pharmacogenomics of human OATP transporters. *Naunyn Schmiedebergs Arch. Pharmacol.* Mar 2006;372(6):432-443.
30. Donnelly LA, Doney AS, Tavendale R, et al. Common nonsynonymous substitutions in SLCO1B1 predispose to statin intolerance in routinely treated individuals with type 2 diabetes: a go-DARTS study. *Clin. Pharmacol. Ther.* Feb 2011;89(2):210-216.
31. Puccetti L, Ciani F, Auteri A. Genetic involvement in statins induced myopathy. Preliminary data from an observational case-control study. *Atherosclerosis.* Jul 2010;211(1):28-29.
32. Brunham LR, Lansberg PJ, Zhang L, et al. Differential effect of the rs4149056 variant in SLCO1B1 on myopathy associated with simvastatin and atorvastatin. *Pharmacogenomics J.* Jun 2012;12(3):233-237.
33. Ramsey LB, Johnson SG, Caudle KE, et al. The Clinical Pharmacogenetics Implementation Consortium (CPIC) guideline for SLCO1B1 and simvastatin-induced myopathy: 2014 update. *Clin. Pharmacol. Ther.* Jun 11 2014.
34. Voora D, Shah SH, Spasojevic I, et al. The SLCO1B1*5 genetic variant is associated with statin-induced side effects. *J. Am. Coll. Cardiol.* Oct 20 2009;54(17):1609-1616.
35. Oshiro C, Mangravite L, Klein T, Altman R. PharmGKB very important pharmacogene: SLCO1B1. *Pharmacogenet. Genomics.* Mar 2010;20(3):211-216.
36. Exome Aggregation Consortium. ExAC Browser Beta. 2015; http://exac.broadinstitute.org. Accessed February 20, 2015, 2015.
37. Canestaro WJ, Austin MA, Thummel KE. Genetic factors affecting statin concentrations and subsequent myopathy: a HuGENet systematic review. *Genet. Med.* Nov 2014;16(11):810-819.
38. Whirl-Carrillo M, McDonagh EM, Hebert JM, et al. Pharmacogenomics knowledge for personalized medicine. *Clin. Pharmacol. Ther.* Oct 2012;92(4):414-417.
39. Bielinski SJ, Olson JE, Pathak J, et al. Preemptive genotyping for personalized medicine: design of the right drug, right dose, right time-using genomic data to individualize treatment protocol. *Mayo Clin. Proc.* Jan 2014;89(1):25-33.

40. Karnes JH, Van Driest S, Bowton EA, et al. Using systems approaches to address challenges for clinical implementation of pharmacogenomics. *Wiley Interdiscip. Rev. Syst. Biol. Med.* Mar-Apr 2014;6(2):125-135.
41. Grant RW, O'Brien KE, Waxler JL, et al. Personalized genetic risk counseling to motivate diabetes prevention: a randomized trial. *Diabetes Care.* Jan 2013;36(1):13-19.
42. Vassy JL, O'Brien KE, Waxler JL, et al. Impact of literacy and numeracy on motivation for behavior change after diabetes genetic risk testing. *Med. Decis. Making.* Jul-Aug 2012;32(4):606-615.
43. Vassy JL, McLaughlin HM, MacRae CA, et al. A One-Page Summary Report of Genome Sequencing for the Healthy Adult. *Public health genomics.* Jan 21 2015.
44. Fiore LD, Brophy M, Ferguson RE, et al. A point-of-care clinical trial comparing insulin administered using a sliding scale versus a weight-based regimen. *Clin. Trials.* Apr 2011;8(2):183-195.
45. D'Avolio L, Ferguson R, Goryachev S, et al. Implementation of the Department of Veterans Affairs' first point-of-care clinical trial. *J. Am. Med. Inform. Assoc.* June 1, 2012 2012;19(e1):e170-e176.
46. U.S. Department of Veterans Affairs Office of Research and Development. Point of Care Research (POC-R). 2015; http://www.research.va.gov/services/csrd/point-of-care.cfm. Accessed February 23, 2015, 2015.
47. Weir CR, Butler J, Thraen I, et al. Veterans Healthcare Administration providers' attitudes and perceptions regarding pragmatic trials embedded at the point of care. *Clin. Trials.* Mar 20 2014.
48. Vassy JL, Shrader P, Yang Q, et al. Genetic associations with metabolic syndrome and its quantitative traits by race/ethnicity in the United States. *Metab. Syndr. Relat. Disord.* Dec 2011;9(6):475-482.
49. Vassy JL, Durant NH, Kabagambe EK, et al. A genotype risk score predicts type 2 diabetes from young adulthood: the CARDIA study. *Diabetologia.* Oct 2012;55(10):2604-2612.
50. Vassy JL, Hivert MF, Porneala B, et al. Polygenic type 2 diabetes prediction at the limit of common variant detection. *Diabetes.* Feb 11 2014.
51. Vassy JL, Mahapatra PD, Meigs JB, et al. Genotype predicts type 2 diabetes in adulthood in a multiracial adolescent population. *Pediatrics.* November 2012 2012;130(5):e1235-1242.
52. Christensen KD, Vassy JL, Phillips KA, et al. Short-term costs of integrating whole-genome sequencing into primary care and cardiology settings: a pilot randomized trial. *Genet Med.* 2018.
53. Ramsey SD, Willke RJ, Glick H, et al. Cost-effectiveness analysis alongside clinical trials II-An ISPOR Good Research Practices Task Force report. *Value Health.* 2015;18(2):161-172.
54. Sanders GD, Neumann PJ, Basu A, et al. Recommendations for Conduct, Methodological Practices, and Reporting of Cost-effectiveness Analyses: Second Panel on Cost-Effectiveness in Health and Medicine. *JAMA.* 2016;316(10):1093-1103.
55. Frick KD. Microcosting quantity data collection methods. *Med Care.* 2009;47(7 Suppl 1):S76-81.
56. Glynn RJ, Brookhart MA, Stedman M, Avorn J, Solomon DH. Design of cluster-randomized trials of quality improvement interventions aimed at medical care providers. *Med. Care.* Oct 2007;45(10 Supl 2):S38-43.
57. Pocock SJ. *Clinical Trials: A Practical Approach.* New York: John Wiley & Sons; 1983.
58. Julious SA. Sample sizes for clinical trials with normal data. *Stat. Med.* Jun 30 2004;23(12):1921-1986.
