# Supplementary material for: Effect of Pharmacogenetic Testing for Statin Myopathy Risk vs Usual Care on Blood Cholesterol: A Randomized Clinical Trial
Source: JAMA Netw Open. 2020 Dec 3;3(12):e2027092. doi: 10.1001/jamanetworkopen.2020.27092 (PMC7716196; doi:10.1001/jamanetworkopen.2020.27092)
Supplement: Supplement 2. — eTable 1. Evidence of Statin-Associated Muscle Symptoms (SAMS) in the Integrating Pharmacogenetics in Clinical Care (I-PICC) Study eTable 2. Electronic Health Record (EHR) Documentation and Patient Communication Process Outcomes Among I-PICC Study Patient Participants in the Intervention (PGx+) Arm eTable 3. Patient Beliefs About Medications and Test Results Recall at 12 Months [file jamanetwopen-e2027092-s002.pdf]

Supplemental Online Content

Vassy JL, Gaziano JM, Green RC, et al. Effect of pharmacogenetic testing for statin myopathy risk vs usual care on blood cholesterol: a randomized clinical trial. *JAMA Netw Open*. 2020;3(12):e2027092. doi:10.1001/jamanetworkopen.2020.27092

eTable 1. Evidence of Statin-Associated Muscle Symptoms (SAMS) in the Integrating Pharmacogenetics in Cinical Care (I-PICC) Study
eTable 2. Electronic Health Record (EHR) Documentation and Patient Communication Process Outcomes Among I-PICC Study Patient Participants in the Intervention (PGx+) Arm
eTable 3. Patient Beliefs About Medications and Test Results Recall at 12 Months

This supplemental material has been provided by the authors to give readers additional information about their work.

© 2020 Vassy JL et al. *JAMA Network Open.*

eTable 1: Evidence of Statin-Associated Muscle Symptoms (SAMS) in the Integrating Pharmacogenetics in Cinical Care (I-PICC) Study

	**Intervention (*n*=193)**	**Control (*n*=215)**
Creatine kinase testing, *n* (%)	35 (18.1)	32 (14.9)
Provider-documented SAMS[a], *n* (%)	2 (1.0)	3 (1.4)
Creatine kinase testing among patients with provider-documented SAMS, *n* (%)	2 (100.0)	1 (33.3)
	**Intervention (*n*=169)**	**Control (*n*=203)**
Patient-reported SAMS[b], *n* (%)	3 (1.8)	3 (1.5)

[a]SAMS documentation abstracted from 12-month chart review.
[b]SAMS report assessed at 12-month patient survey (total completed surveys, *n*=372)

© 2020 Vassy JL et al. *JAMA Network Open.*

eTable 2: Electronic Health Record (EHR) Documentation and Patient Communication Process Outcomes Among I-PICC Study Patient Participants in the Intervention (PGx+) Arm

	**PGx+ (*n*=193)**
Results documentation by provider in EHR, *n* (%)	32 (16.6)
Results communication to patient, *n* (%)	30 (15.5)
Office visit, *n* (%)	4 (13.3)
Templated letter, *n* (%)	7 (23.3)
Other letter, *n* (%)	18 (60.0)
Telephone call, *n* (%)	1 (3.3)
Statin allergy documented in structured field, *n* (%)	3 (1.6)

Outcomes observed during the 12 months after enrollment.

© 2020 Vassy JL et al. *JAMA Network Open.*

eTable 3: Patient Beliefs About Medications and Test Results Recall at 12 Months

		PGx+ (*n*=169)	PGx- (*n*=203)
"My health in the future will depend on my medicines"[a]	Strongly agree	28 (16.6)	30 (14.8)
	Agree	65 (38.5)	84 (41.4)
	Uncertain	29 (17.2)	37 (18.2)
	Disagree	29 (17.2)	40 (19.7)
	Strongly disagree	17 (10.1)	12 (5.9)
"Medicines do more harm than good"[a]	Strongly agree	9 (5.3)	10 (4.9)
	Agree	26 (15.4)	20 (9.9)
	Uncertain	46 (27.2)	56 (27.6)
	Disagree	63 (37.3)	89 (43.8)
	Strongly disagree	24 (14.2)	28 (13.8)
Recall of PGx testing, *n* (%)		11 (6.5)	
Correct results recall, *n* (%)[b]		2 (18.2)	

Abbreviations: PGx, pharmacogenetic; PGx+, intervention arm; PGx-, control arm.
[a]One survey PGx+ participant did not respond to this question.
[b]Among participants reporting that they recalled undergoing genetic testing, the number who correctly recalled the interpretation of their *SLCO1B1* results.

© 2020 Vassy JL et al. *JAMA Network Open.*
