# Supplementary material for: Effect of Pharmacogenetic Testing for Statin Myopathy Risk vs Usual Care on Blood Cholesterol: A Randomized Clinical Trial
Source: JAMA Netw Open. 2020 Dec 3;3(12):e2027092. doi: 10.1001/jamanetworkopen.2020.27092 (PMC7716196; doi:10.1001/jamanetworkopen.2020.27092)
Supplement: Supplement 3. — Data Sharing Statement [file jamanetwopen-e2027092-s003.pdf]

# Data Sharing Statement

Vassy. Effect of Pharmacogenetic Testing for Statin Myopathy Risk vs Usual Care on Blood Cholesterol. *JAMA Netw Open*. Published December 03, 2020. 10.1001/jamanetworkopen.2020.27092

**Data**
**Data available:** Yes
**Data types:** Deidentified participant data, Data dictionary
**How to access data:** Data will be available in a data repository accessible at the following URL: https://www.vacsp.research.va.gov/CSPEC/Studies/INVESTD-R/Integrating-Pharmacogenetics-clinical-care-study.asp
**When available:** With publication

**Supporting Documents**
**Document types:** Statistical/analytic code, Informed consent form
**How to access documents:** Documents will be available in a data repository accessible at the following URL: https://www.vacsp.research.va.gov/CSPEC/Studies/INVESTD-R/Integrating-Pharmacogenetics-clinical-care-study.asp.
**When available:** With publication

**Additional Information**
**Who can access the data:** Data will be made available to researchers whose proposed use of the data has been approved.
**Types of analyses:** Data will be made available for non-commercial purposes.
**Mechanisms of data availability:** Data will be made available with investigator support after approval of a proposal.
